# Anxiety at outpatient hysteroscopy

**DOI:** 10.1007/s10397-015-0895-3

**Published:** 2015-05-13

**Authors:** Pietro Gambadauro, Ramesan Navaratnarajah, Vladimir Carli

**Affiliations:** Karolinska Institutet, LIME/NASP-C7, 17177 Stockholm, Sweden; Res Medica Sweden, Uppsala, Sweden; St. Bartholomew’s and the Royal London Hospital, Bart’s Health NHS Trust, Queen Mary University of London, London, UK; WHO Collaborating Centre for Research, Training and Methods Development, and National Centre for Suicide Research and Prevention of Mental lll-Health, Karolinska Institutet, Stockholm, Sweden

**Keywords:** Hysteroscopy, Anxiety, Pain, Outpatient hysteroscopy, Mental health, Patient-centred care

## Abstract

This review summarises current understanding and research on the association between anxiety and outpatient hysteroscopy. Women undergoing hysteroscopy suffer from significant levels of anxiety, with repercussions on pain perception, success rates and satisfaction. Using validated tools such as the Spielberger State-Trait Anxiety Index (STAI) or the Hospital Anxiety and Depression Scale (HADS) in the outpatient hysteroscopy setting, average state anxiety scores similar or greater than those measured before more invasive procedures under general anaesthesia have been consistently reported. This clearly suggests a significant gap between our clinical viewpoint of what is “minimally invasive” and patients’ expectations. In spite of its potential role of confounder in studies on pain-reduction interventions, we found that patient anxiety was evaluated in only 9 (13 %) out of a sample of 70 randomised controlled trials on outpatient hysteroscopy published since 1992. Factors such as trait anxiety, age, indication and the efficiency of the clinic can be correlated to state anxiety before hysteroscopy, but more robust data are needed. Promising non-pharmacological interventions to reduce anxiety at hysteroscopy include patient education, communication through traditional or multimedia approaches, interaction and support during the procedure and music listening.

## Introduction

During the last decades, enormous progress in technique and instruments has turned hysteroscopy into a common outpatient procedure, with both diagnostic and therapeutic potential, and increasing patient compliance [1, 2]. The assumption of the minimal invasiveness of hysteroscopy is based on facts such as the miniaturisation of scopes, the uncommon need of anaesthesia and the progressive simplification of the technique [3, 4]. This is certainly true, also when considering the burden of predecessors such as dilatation and curettage (D&C) or laparotomy. However, the common professional interpretation of what is “minimally invasive” does not take into account a patient’s emotional experience while many women undergoing hysteroscopy suffer from significant levels of anxiety [5, 6].

The aim of this review was to summarise the current understanding and research on the association between anxiety and outpatient hysteroscopy. A secondary objective was to determine whether published randomised trials reporting pain at hysteroscopy to date have considered anxiety as a confounding factor.

## Methods

We searched scientific literature on anxiety related to hysteroscopy from PubMed, Scopus and PsychINFO online databases, using the keywords “anxiety” and “hysteroscopy” including thin word variants. We then searched reference lists of known systematic reviews [7–14] and PubMed for randomised controlled trials reporting pain at outpatient hysteroscopy. On PubMed we used a query based on the keyword “hysteroscopy” and the filter “randomized controlled trial [ptype]”. This simple query has got high sensitivity (93.7 %; 95 %CI 92.5–94.9) and specificity (97.6 %; 95 %CI 97.4–97.7) for the identification of randomised trials on PubMed [15].

A total of 304 abstracts were considered for a general review on various specific aspects of anxiety at outpatient hysteroscopy, such as prevalence, intensity, risk factors and management. When the material found on hysteroscopy was deemed insufficient, general literature on preoperative anxiety in gynaecology, or other surgical disciplines, was reviewed.

We also conducted a targeted analysis aimed at investigating whether published trials on pain at hysteroscopy control for patient anxiety levels. The articles included in this analysis were selected according to the following criteria: being a RCT, hysteroscopies performed in an outpatient setting and pain as a primary or secondary outcome. Exclusion criteria were the following: trials where hysteroscopy was compared to other procedures such as operative hysteroscopy or ultrasound, pain not measured as an outcome and patients receiving general anaesthesia. Data were extracted regarding year of publication, study intervention (e.g. anaesthetic agent, instrumentation or distension media), assessment of patient anxiety and, in the latter case, tools used for measurement.

The findings of this comprehensive review are presented through the following three paragraphs, dealing respectively with the relevance of pre-hysteroscopy anxiety, its prevalence and intensity and possible management. A narrative format was chosen as a result of very heterogeneous data.

## Findings

### Anxiety at hysteroscopy: irrelevant or underestimated?

Anxiety is defined as an “abnormal and overwhelming sense of apprehension and fear often marked by physiological signs, doubt concerning the reality and nature of the threat, and by self-doubt about one’s capacity to cope with it” [16]. Some anxiety connected to stress situations is in many cases inevitable. However, intense symptoms of anxiety might be related to personality characteristics and interfere with everyday activities, with negative health-related consequences.

Patient anxiety in association with a medical encounter, is a well-known phenomenon whose possibly most recognised and studied manifestation is the “white coat effect” [17]. Within healthcare, surgery represents the archetypal invasive method of managing disease, which reflects on the well-known high levels of preoperative anxiety commonly reported in the literature [18]. Surgery-related anxiety is an important problem that has also got negative repercussions and consequences before and after the procedure. Sleep disturbances, for instance, are common before gynaecological endoscopic surgery, regardless of the nature and extent of the operations [19]. Anxiety is a possible risk factor for postoperative nausea and vomiting [20]. Preoperative anxiety prior to gynaecological surgery is associated with increased and persistent postoperative pain [21, 22]. Anxiety before major procedures can be triggered by the intrinsic invasiveness and risks of surgery and by an understandable fear of the loss of control linked to anaesthesia. One would expect lower anxiety levels before minimally invasive or outpatient procedures, which are very well represented in gynaecology. Hysteroscopy is one of the most emblematic minimal access gynaecological procedures. Initially, it replaced other more invasive techniques to become a routine outpatient procedure performed with minimal or no anaesthetic requirements [4, 23]. Paradoxically, despite the evolution of scope technology, intense preoperative anxiety has been reported in earlier [24] as well as in recent years of hysteroscopic development [25]. This suggests that the patient’s perception of this common procedure induces anxiety, regardless of how patient-friendly hysteroscopy has become.

Anxiety can have consequences which are specific to hysteroscopy when performed in an outpatient setting on conscious patients, above all pain [5, 6]. Patients might experience pain as a result of physical stimuli such as cervical dilatation, intrauterine pressure and manipulation. However, pain is subjective and multifactorial, and its perception is modulated by mood and emotional states such as anxiety [26]. This explains why similar physical stimuli lead to great differences in pain perception and justify the finding that non-organic factors such as anxiety predict pain at hysteroscopy [5, 6].

In this context, we would expect that patient anxiety levels would be assessed in clinical trials on pain reduction interventions at outpatient hysteroscopy. In order to test this hypothesis, we have systematically reviewed relevant literature with the aim to assess whether published RCTs on outpatient hysteroscopy considered patients’ anxiety as a variable. As summarised in Table 1, through our search strategy, we have identified 70 RCTs published from 1992 to 2013. Pre-hysteroscopy anxiety levels were evaluated in only 9 studies (13 %). Interestingly, a single research group (Adam Magos, London, UK) authored almost half of those articles (4/9). Therefore, published research on pain at hysteroscopy has not taken anxiety as a potential confounding factor until now, and this might constitute a significant bias.

**Table 1:** How often is anxiety assessed in RCTs on pain reduction interventions at outpatient hysteroscopy?

		Number	Percentage
RCTs included in analysis		70	100
Period	1992–2002 2003–2013	25 45	35.7 64.3
Intervention	Anaesthesia/analgesia Instruments Cervical preparation Distension media Technique Others	32 9 9 9 8 3	45.7 12.9 12.9 12.9 11.4 4.3
Pain as main endpoint	Yes No	57 13	81.4 18.6
Anxiety assessment		9	13
	Method of assessment		
	Single question^a^ VAS POMS^b^ STAI Unclear	2 4 1 1 1	22 44 11 11 11

^a^Yes/no or quiet/anxious?

^b^Profile of Mood States

Obviously, diagnostic hysteroscopy is a relatively short procedure, and the possibility of undergoing a quick, outpatient procedure is preferred by most patients, who might accept experiencing some pain or discomfort instead of waiting for an anachronistic procedure under general anaesthesia [27]. Nonetheless, pain is not just a problem in itself since it correlates to the success of outpatient hysteroscopy, both diagnostic and operative [28, 2].

Anxiety, apart from increasing pain perception and the risk of failure of an outpatient hysteroscopy, also seems to determine lower patient satisfaction [29]. As previously reported, patients with higher level of anxiety would be more likely to choose general anaesthesia if they needed to undergo a new hysteroscopy in the future [29]. We believe that this information is extremely relevant since patient satisfaction is crucial in the context of modern patient-centred care. We could not find any data on the long-term effects of anxiety on satisfaction after hysteroscopy, but experiences on patients undergoing insertion of intrauterine devices show that women with higher pre-procedural anxiety still remember the procedure as a painful experience after 6 months [30].

### Prevalence and intensity of anxiety at hysteroscopy

Elevated levels of anxiety in patients waiting for hysteroscopy have been reported in the last decades. In a large Italian study published in 2007, 65 % of 533 women interviewed by a physician before office hysteroscopy reported preoperative anxiety, defining it as “an unpleasant state of uneasiness or tension” [6]. However, measuring anxiety by answering a direct question (e.g. “do you feel anxious?”) might lack validity, and therefore, other authors have attempted to measure pre-hysteroscopic anxiety by other structured, validated methods.

Dickson and Depares, back in 2000, published their measurements of anxiety levels among 30 women before outpatient hysteroscopy [24]. The Spielberger State-Trait Anxiety Index (STAI) was used, and an average anxiety level of 46.07 (±11.39 SD) was detected. The STAI is a self-administered, 40-item questionnaire consisting of two parts (S as state, and T as trait), which was first introduced in the 1970s, and revised in 1983 [31, 32]. One of its peculiarities is the ability to differentiate a present anxiety state (STAI-s; 20 items) from a long-standing trait anxiety (STAI-t; 20 items). Scores range from 20 (minimum anxiety) to 80 (maximum anxiety). It is relatively quick to complete (around 10 min) and is available in many languages. A short version of the STAI-s (6 items) is available [33].

The STAI has often been used as a measure of anxiety in patients undergoing hysteroscopy.

A study from 2004 reported STAI-measured state anxiety levels in 240 women attending an outpatient see-and-treat hysteroscopy clinic and compared them to women in other clinical situations [29]. The mean anxiety levels measured were 45.7 (median 45) at a full, 20-item STAI, and 47.3 at a reduced, 6-item STAI. Anxiety levels before hysteroscopy were significantly higher than those measured among 73 women attending a general gynaecology clinic (median 39, *p* = .004), while similar levels were detected among 36 women seen at a chronic pelvic pain clinic (median 46).

More recently, similarly high anxiety levels before hysteroscopy were confirmed by other authors. Carta et al. reported median STAI-s values of 41.50 (range 20–73) in a sample of 94 women [34]. In this study, 80 % of the women had moderate to severe anxiety state, defined by the authors as a STAI-s value ≥34. In the context of an RCT on the effect of music on pain, STAI-s scores of 39.45 were measured in a study population of 356 women undergoing office hysteroscopy [25]. In a very recent study evaluating the impact of anxiety on pain at hysteroscopy, the mean pre-procedural STAI-s score of 148 women was 44.8 ± 10.0 (SD) [5].

High anxiety levels have also been reported few days before outpatient hysteroscopy, outside the hospital environment. Tarling et al. used a 6-item STAI-s to assess anxiety in 18 postmenopausal patients receiving a priority referral to diagnostic hysteroscopy in the context of an urgent assessment of postmenopausal bleeding [35]. Eighteen patients were mailed the questionnaire home 4 days after the first encounter with the specialists, hence while waiting for hysteroscopy. These women had mean anxiety levels of 43.89 ± 17.98 (SD) compared to levels of 38.96 ± 9.79 (SD) among 16 women undergoing the same urgent assessment, but not referred to hysteroscopy.

The Hospital Anxiety and Depression Scale (HADS) is another validated tool which was designed to specifically screen patients for anxiety and depression. It can be self-administered. Its A component (HADS-A) focuses on anxiety and consists of 7 items. Scores might range between 0 and 21, with values below eight interpreted as normal (no anxiety). Within the setting of endometrial and ovarian cancer screening in 26 women with family history of hereditary non-polyposis colorectal cancer, Wood et al. reported mean values of 6.8 ± 4.2 (95 %CI 5.2–8.5) at HADS anxiety subscale, before combined assessment with hysteroscopy and biopsy, transvaginal ultrasound and blood sampling for CA125 [36]. In this study, the patients were asked to fill the questionnaire at home and then bring it to the visit. Although the recorded scores are considered within the normal range (<8), they are higher than in women before laparoscopic tubal ligation (HADS-A median score 4) and similar to those before hysterectomy (HADS-A median score 7) [37, 22].

A simple 1 to 10 visual analog scale (VAS) has also been used by some authors. As we found in our review of RCTs on pain at hysteroscopy, the VAS was the most used tool to verify that anxiety levels between cases and controls were not statistically different (Table 1). In a cohort of 77 women undergoing hysteroscopy, Mc Gurgan et al. reported VAS measured anxiety scores of 3.5–4 (range 0.5–10), which, however, is a finding of difficult interpretation since scarce control data are found in the literature [38]. For instance, a 100-mm VAS has been validated for preoperative anxiety in the field of dental care, where authors have found a significant correlation with STAI scores, with a reference threshold for anxiety around 50 mm (corresponding to STAI-s 40) [39]. In gynaecology, Hong et al. found mean VAS of 4.7 ± 2.3 among women undergoing egg collection, and the corresponding STAI-s scores in the same population were 40.0 ± 9.7 [40].

Table 2 summarises the results of measurements that have been used to evaluate anxiety before outpatient hysteroscopy together with reference values obtained by published literature in the field of gynaecological surgery. Interestingly, women undergoing outpatient hysteroscopy appear to have higher levels than women undergoing laparoscopic tubal ligation [37] (Table 2; footnotes). On the contrary, the anxiety experienced before hysteroscopy is comparable to that by women undergoing gynaecological surgery under general anaesthesia [22, 41] (Table 2; footnotes). This clearly suggests a significant gap between our clinical viewpoint of what is minimally invasive and the patient’s expectation about an outpatient hysteroscopy procedure.

**Table 2:** Measuring anxiety before outpatient hysteroscopy

Measurement	Scoring range	Author	Number	Age	Values
Patient interview	Yes or no	Cicinelli et al. 2007 [6]	533	n/a	65 %^a^
VAS	1–10	Mc Gurgan et al. 2001 [38]	77	48^b^	3.5–4^b^
HADS-A	0–21	Wood et al. 2008 [36]	26	42.27^c^	6.8^c^
STAI-s	20–80	Dickson & Depares 2000 [24]	30	n/a	46.07^c^
		Gupta et al. 2004 [29]	240	n/a	45^b^
		Carta et al. 2012 [34]	94	48^b^	41.5^b^
		Angioli et al. 2014 [25]	356	56.05^c^	39.45^c^
		Kokanali et al. 2014 [5]	148	43.6^c^	44.8^c^
6-item STAI-s	20–80	Gupta et al. 2004 [29]	240	n/a	47.3^c^
		Tarling et al. 2013 [35]	18	63^c^	43.15^c^

Values were obtained on the day of hysteroscopy in all studies except for two of them [35, 36] where patients were sent the questionnaire by post. HADS-A median score before laparoscopic tubal ligation, 4 (59 women) [37]; before hysterectomy, 7 (186 women) [22]. STAI-s median score before laparoscopic tubal ligation, 29 (59 women) [37]; before gynaecological surgery, 42 (45 women) [41]

^a^Percentage of women answering “yes”

^b^Median score

^c^Mean score

### Can anxiety before hysteroscopy be predicted or reduced?

We have documented the high levels of pre-hysteroscopy anxiety in the previous paragraphs and their potential impact on patient well-being and the success of the procedure. On the basis of this background, efforts should be made in order to identify predictors or interventions that could help respectively in identifying subjects at higher risk for anxiety and in preventing/limiting anxiety and its consequences.

#### Which factors are correlated to anxiety before hysteroscopy?

We will first summarise the knowledge we have about factors associated to anxiety. It would be logical to expect people with a trait anxiety to be more vulnerable to preoperative stress and therefore likely to experience higher levels of state anxiety before hysteroscopy. State anxiety is correlated to situations such as waiting for an invasive procedure like hysteroscopy. Trait anxiety predisposes some individuals to experience more anxiety states than others, both in frequency and intensity [42]. It also appears to be associated with ethnic and racial factors [43]. This knowledge would justify using both STAI questionnaires in order to control for trait anxiety. Interestingly, in one of the largest studies on pre-hysteroscopy anxiety reviewed here, the authors decided not to measure trait anxiety, stating that “there was no reason to think that these people were more anxious than any others in a general population” [29]. This assumption, albeit reasonable, does not seem to be unequivocally confirmed by the available literature (Table 3). Trait anxiety among women undergoing office hysteroscopy appears to be common, and it also independently predicts pain during the procedure as well as 60 min after, as recently reported by Kokanali et al. [5]. Further studies would be needed to verify whether women referred to hysteroscopy are more likely to have anxiety traits and whether an anxiety trait is directly correlated to state anxiety in the same women.

**Table 3:** Trait anxiety in women undergoing outpatient hysteroscopy

Author	Year	Number	Age	STAI-t average score
Carta et al. [34]	2012	94	48^a^	38^a^
Angioli et al. [25]	2014	356	56.05^b^	35.9^b^
Kokanali et al. [5]	2014	148	43.6^b^	38.4^b^

STAI-s median score before laparoscopic tubal ligation, 30 (59 women) [37]; before gynaecological surgery, 39 (45 women) [41]

^a^Median

^b^Mean

Age is another factor potentially correlated with anxiety. Although hysteroscopy may be needed at almost any age, it is more common in middle-aged women (see column “age” in Table 2). The frequent association of perimenopause with abnormal uterine bleeding should be taken into account when studying anxiety in these patients. As a matter of fact, recent research shows that women with previous low levels of anxiety become susceptible to higher levels of anxiety once transiting through the menopause [44].

As hysteroscopy is a gynaecological procedure, gender cannot be used as an independent variable when studying preoperative anxiety. Nevertheless, it is worth mentioning that anxiety is more common among women [45]. This also reflects on situational anxiety before surgery. Several studies have demonstrated that preoperative anxiety, regardless of the operation or the anaesthetic strategy, is significantly more frequent as well as more intense among women [46–48]. When it comes to the specific case of outpatient hysteroscopy, the gender of the gynaecologist has been studied in relation to patient anxiety. Reassuringly, Mc Gurgan et al. did not find any statistical difference in anxiety levels in women, whether they were attended by male (VAS 4; range 0.5–10) or female (VAS 3.5; 0.5–9) gynaecologists [38].

The indication to hysteroscopy can certainly correlate with patient anxiety although this hypothesis has not been thoroughly tested. We could, for instance, postulate that women fearing a cancer diagnosis would be more anxious [49]. A study from 2008 failed to demonstrate significant anxiety levels in women undergoing gynaecological cancer screening for hereditary colorectal cancer [36]. More recently, Tarling et al. reported anxiety levels among women undergoing hysteroscopy within an urgent assessment of postmenopausal bleeding [35]. Although these women had high anxiety scores at a 6-item STAI questionnaire (43.15 ± 18.35), those were not significantly higher than in women undergoing the same urgent assessment, but exempted from hysteroscopy (38.96 ± 9.79) [35].

An operative hysteroscopy could have a greater impact on anxiety compared to a diagnostic procedure. Nevertheless, no significant differences in pre-hysteroscopy anxiety were reported by an Italian group of researchers comparing patients undergoing diagnostic and operative office hysteroscopy [25]. The authors, however, did not report whether the procedures were planned or performed according to a see-and-treat protocol. In the case of see-and-treat procedures, the patients would not have had awareness of the operative nature of the upcoming hysteroscopy at the time anxiety was measured.

Interestingly, a higher grade of concern before hysteroscopy has been reported among infertile women [50]. In that study, pain was also more frequent among the same infertile women, although a formal assessment of their anxiety levels was not performed.

Factors not directly linked to the patients can cause anxiety before hysteroscopy. Carta et al. found that having to wait 60 min or more for the procedure is associated to a higher likelihood of pain (OR 5.67; 95 %CI 1.48–37.39) [34]. Although the waiting time in that study seemed to be exceptionally long (median 100 min; range 20–420), similar findings were more recently confirmed by Kokanali et al., who found in-hospital waiting time to be significantly correlated to pain scores during hysteroscopy as well as 60 min after [5]. Long waiting lists before the procedure may also interfere with patients’ experience. This has not been reported for hysteroscopy, but it is suggested by studies in other settings. For instance, a shorter waiting list for prostate biopsy appears to increase patient tolerance of the procedure, particularly in the case of higher anxiety [51]. The cited experiences support the idea that a good healthcare organisation improves patient experience.

#### How would anxiety before hysteroscopy be managed best?

In the inpatient surgery setting, preoperative anxiety is commonly managed by means of pharmacological interventions such as the use of anxiolytics and sedatives. Some authors describe the routine administration of anxiolytics in the setting of outpatient hysteroscopy under local anaesthesia [52, 53]. However, in view of the commonly short duration of the procedure, and the possible side effects of oral medication, non-pharmacological tools would be preferable and more in line with the minimalistic philosophy of modern office hysteroscopy.

Communication and patient education have been proved as effective tools to reduce preoperative anxiety, and their role should be increasingly acknowledged, now that many patients are active consumers of unfiltered and largely unreliable information through the Internet. It has been demonstrated that preoperative anxiety is effectively reduced by the ability of doctors to answer patients’ questions, which also increases patients’ satisfaction [54]. Clarity is more important than the amount of information in order to reduce perceived anxiety before gastrointestinal endoscopy [55]. This might explain why standard written information is appreciated by patients, and it does not increase their anxiety [56]. Multidisciplinary strategies, involving psychological interventions, have been advocated in order to reduce anxiety in complex cases with high preoperative anxiety [57]. The difficulty of an effective preoperative communication could be overcome by multimedia approaches which seem to improve patient understanding and satisfaction, although a significant effect on preoperative anxiety has not been yet confirmed [58].

Since women are awake at outpatient hysteroscopy, communication is not just limited to preoperative setting but can continue during the procedure. Morgan et al. have studied the experiences and attitudes of women undergoing hysteroscopy [27]. According to their results, patients are positive about receiving continuous information on the progress of the procedure and on what they should expect. Many women also chose to follow the hysteroscopy on the screen because they were either interested or thought that watching could help them focus on something else. But almost half of the women chose not to look at the screen [27]. This might justify the results reported by authors who found that watching the screen during hysteroscopy is not beneficial to patients [59]. Interestingly, in the neighbouring field of colposcopy, recorded and live videos can significantly reduce patient anxiety [60–62]. Considering both the available literature and our own experience with surgical-video-mediated patient education, we would argue that the possibility to follow one’s own hysteroscopy live on a screen might improve the interaction between patient and caregivers in many cases, although the choice of whether to look or not should be left to the patient [63, 64].

An improvement in pain thresholds and vaginal birth rates has been reported in obstetric research as a result of patient support by friends or “doulas” [65]. Similarly, some evidence shows that a relevant role in surgery-related anxiety reduction is played by nurses and nurse practitioners [66]. It would be useful to attempt replicating those findings in the hysteroscopic setting.

Finally, an interesting and possibly cost-effective tool to reduce preoperative anxiety is represented by music listening. Music interventions are known to have a general positive effect on anxiety reduction in medical patients [67]. A recent systematic review has demonstrated how effective listening to music can be in terms of preoperative anxiety reduction [68]. This has also been shown in the specific setting of day surgery, endoscopy and colposcopy [62, 69–71]. A recent randomised trial in office hysteroscopy has shown that listening to music during hysteroscopy might reduce pain, possibly as a consequence of a reduction of anxiety [25]. The question whether music might also reduce anxiety when listened prior to hysteroscopy remains open.

## Conclusions

Women undergoing outpatient hysteroscopy suffer from significant levels of preoperative anxiety, comparable to those experienced before major surgery under general anaesthesia.

This can have repercussions on pain perception, success of the procedure as well as on overall patient experience and satisfaction. Anxiety, to date, has rarely been evaluated as a confounding factor in published RCTs reporting pain at outpatient hysteroscopy. In the future, similar randomised trials should measure preoperative anxiety with validated tools, in order to reduce the risk of significant bias. Factors such as trait anxiety, age, indication and the efficiency of the clinic can be correlated to state anxiety before hysteroscopy, but more robust data are needed. Promising non-pharmacological interventions to reduce anxiety at hysteroscopy include patient education, communication through traditional or multimedia approaches, interaction and support during the procedure and music listening.
